# Supplementary material for: Spectral indices with different spatial resolutions in recognizing soybean phenology
Source: PLoS One. 2024 Sep 18;19(9):e0305610. doi: 10.1371/journal.pone.0305610 (PMC11410218; doi:10.1371/journal.pone.0305610)
Supplement: Attachment — Submitted filename: Response to Reviewers.docx [file pone.0305610.s001.docx]

**Response to Reviewers**

Regarding Figure 1, the satellite image obtained from Google Earth was replaced by an image collected by the CBERS4A satellite, a platform belonging to the Brazilian State that, like the LANDSAT system, has free distribution without copyright restrictions.

**Reviewer #1:**

1. The goal of the study is not clear to mean, or have not been well articulated in the last paragraph of the introduction. In fact, what the key research questions that your work seek to address?

The aim of the research was to evaluate the efficiency of different vegetation indexes, obtained from satellites with different spatial resolutions, in discriminating the phenological stages of soybean crops. We made the necessary changes to the original material to meet the reviewer's recommendations.

2. The methodology is too shallow and has no debt to allow readers identify the novelty or contribution of the work. So much effort was spent on describing the data used, even in the aspect that talked about “Conducting the experiment.” Readers are expecting to see a step-by-step description of how the experiment was conducted. It was difficult to connect flowchart in Figure 1 with the method description. Every aspect of the work is just mixed up with content that ought to be in different section. In this current form, the work lack is no logical coherence. For example, I see you have two figures with caption as Figure 1. Secondly, for the second Figure 1 (flowchart), how was the reprojection done for both satellite images? How was the field data corroborated with the satellite image using the Anderson Discriminant Analysis? Lastly, what do you mean by Conclusions? You flowchart is supposed to be a concise summary of the approach adopted. But here, you confuse readers with “Conclusions”. How are we supposed to understand that while reading your methodology? Please, streamline the methodology to allow for easy reproducibility.

After the review, figure 1 now corresponds to the study location and the methodology flowchart becomes figure 4.

The reprojection of images was only necessary for Sentinel-2 images that are originally available in the WGS 84 coordinate reference system, requiring reprojection to Datum Sirgas 2000 UTM Zone 23S, which corresponds to the UTM projection zone of the study area.

Data collected in the field were correlated with satellite images, both representing the same sampling moment, and were used to verify the possibility of identifying a certain phenological stage, without the need to go to the field. To investigate this possibility, Anderson's Discriminant Analysis was applied to the satellite images and, after corrections, the Neural Networks technique was also applied.

The term “Conclusions” in the image referred to the study conclusions, but, in reviewing the image, the term was replaced by an icon that represents the interpretation of the obtained results.

All corrections, suggested by the reviewer for the methodology, were accepted.

3. While reading your conclusion, it was very difficult to tell if the work actually addressed the goal of the study. I suggest you overhaul the entire manuscript, and then, clearly state the goal or objectives of the study. Then, try to show us in the conclusion that the goal has been achieved.

The conclusions were rewritten to be well adjusted to the objective, as suggested by the reviewer.

Minor comment

1. Why did you hyphenate “recogniz-ing” in the title (line 2)?

Hyphen removed from the title

2. The title of this manuscript can be improved. Think about it carefully.

Suggestion accepted.

3. What do you mean by “Search Location” in line 100? Note that "Search location" and "Study location" are not exactly the same.

The terms have been standardized.

4. It would be very nice to include some statistical findings in your abstract.

Suggestion accepted.

**Reviewer #2:**

1. Title: Spectral indexes with different spatial resolutions in recogniz-ing soybean phenology. The title is not correct. "Indices" is the plural form of "index" when used in the context of measurements or indicators. Secondly the use of the hyphen in ‘recogniz-ing’ is unnecessary in this context. Similar mistake is also repeated in the Abstract section too, like: ‘cul-tivation’, and ‘demon-strates’. Therefore, the accurate title for the paper would be: "Spectral Indices with Different Spatial Resolutions in Recognizing Soybean Phenology." This usage adheres to standard scientific terminology in the field of remote sensing and geographic information systems.

The reviewer's suggestion was fully met.

2. In the abstract section, the findings are verbally expressed without substantiating the same with data. This is not a standard practice. The authors should have briefly described with data (in the abstract section) how different indices are correlated in identifying phonological stages of the soybean crop.

The reviewer's suggestion was fully met.

3. The spatial resolution of Amazonia 1 and sentinel 2 for (NIR, RGB) are 60 m and 10 m respectively. For a better accuracy assessment, the efficiency of each spectral indices needs to be checked at the same spatial resolution for both Amazonia 1 and sentinel 2 imageries, using resampling technique. This can be done either by upscaling the 10 m to 60 m or downscaling the 60 m to 10 m resolution. The authors have ignored this fact.

In this work, the authors chose not to apply the resampling technique in order to simulate the real conditions of data use. So, when a user uses satellite image data, products are generated from images with their original spatial resolution, without resampling. Thus, it is possible to infer the influence of spatial resolution on the accuracy of vegetation indexes in identifying soybean phenological stages.

4. The temporal resolution i.e. the revisit frequency of each single SENTINEL-2 satellite is 10 days while the combined constellation revisit time is 5 days. The authors seem to have confused the revisit time of Sentinel 2 constellation with the temporal variation of MSI sensor onboard Sentinel-2. This is a major correction and it will impact the findings of this study.

The reviewer's suggestion was fully met.

**Reviewer #3:**

1. Consideration of Additional Statistical Tests: While Anderson's discriminant analysis is valuable for assessing classification accuracy, incorporating additional statistical tests such as ANOVA or pairwise comparisons could provide further insights into the significance of differences between vegetation indexes and phenological stages. This would strengthen the statistical robustness of your findings.

ANOVA was performed to identify differences between vegetation indexes at different phenological stages. However, the authors decided to present only the significance of its test (F Test) in figure 5. It was decided not to perform a pairwise comparison test, and, to represent the index values difference in the different phenological stages, it was chosen the line graph shown in figure 5.

2. Exploratory Data Analysis for Variability: Prior to conducting discriminant analysis, performing exploratory data analysis to understand the variability within vegetation indexes across different phenological stages could be beneficial. Box plots or histograms could help visualize the distribution of index values and identify any outliers or trends that may impact the analysis.

Before the ANOVA, box plot graphical analysis was performed to identify outliers, which were eliminated when identified. However, it was decided not to present such graphics in order to make the reading of the work more objective and fluid.

3. Assessment of Model Assumptions: Verify the assumptions underlying Anderson's discriminant analysis, such as multivariate normality and homogeneity of covariance matrices, through diagnostic tests or graphical methods. Addressing violations of these assumptions ensures the reliability of the classification results.

The reviewer's suggestion was fully met.

4. Validation Techniques for Model Performance: Consider employing cross-validation or bootstrap resampling techniques to validate the performance of the discriminant analysis model. This would assess the generalizability of the classification results and provide confidence in the effectiveness of the selected vegetation indexes for phenological stage identification.

The cross-validation technique was applied to the Neural Network Analysis that was added to the study.

5. Comparison with Baseline Models: In addition to evaluating the efficiency of vegetation indexes, compare the performance of the discriminant analysis model with baseline classifiers such as logistic regression or support vector machines. This comparative analysis would offer a broader perspective on the suitability of different statistical approaches for phenological stage classification.

The reviewer's suggestion was fully met and the data was also subjected to Neural Network Analysis. This technique is capable of identifying non-linear patterns in the data set and thus maximizing the identification of groups of samples.

6. Sensitivity Analysis for Model Parameters: Conduct sensitivity analysis to examine the impact of different parameters or settings on the results of Anderson's discriminant analysis. This analysis would help identify optimal parameter choices and enhance the reproducibility of the classification outcomes. By incorporating these suggestions, you can enhance the rigor and validity of your statistical analysis, providing a more comprehensive assessment of the efficiency of vegetation indexes in distinguishing soybean phenological stages.

Specifically, for the suggestion of carrying out sensitivity analyzes to examine the impact of different parameters or configurations on the results of Anderson's discriminant analysis, the data were subjected to quadratic discriminant analysis, however this presented lower representation than the linear one, present in the article. Therefore, the authors decided not to present these results in the manuscript.
